# Predictors of Lymph Node Metastasis in T1 Colorectal Cancer in Young Patients: Results from a National Cancer Registry

**DOI:** 10.3390/jcm10235511

**Published:** 2021-11-25

**Authors:** Daryl Ramai, Jameel Singh, Antonio Facciorusso, Saurabh Chandan, Banreet Dhindsa, Amaninder Dhaliwal, Barbara Galassi, Gianluca Tomasello, Michele Ghidini

**Affiliations:** 1 Division of Gastroenterology and Hepatology, University of Utah, Salt Lake City, UT 84132, USA; 2 Department of Internal Medicine, Mathers Hospital, Port Jefferson, New York, NY 11777, USA; jameel.k.singh@gmail.com; 3 Section of Gastroenterology, Department of Medical Sciences, University of Foggia, 71122 Foggia, Italy; antonio.facciorusso@virgilio.it; 4 Division of Gastroenterology & Hepatology, CHI Health Creighton University Medical Center, Omaha, NE 68124, USA; saurabhchandan@gmail.com; 5 Division of Gastroenterology and Hepatology, University of Nebraska Medical Center, Omaha, NE 68198, USA; banreet.dhindsa@unmc.edu; 6 Division of Gastroenterology, Moffitt Cancer Center, University of South Florida, Tampa, FL 33612, USA; dramaninderdhaliwal@gmail.com; 7 Operative Unit of Medical Oncology, Fondazione IRCCS Ca’ Granda Ospedale Maggiore Policlinico, 20122 Milan, Italy; barbara.galassi@policlinico.mi.it (B.G.); gianluca.tomasello@policlinico.mi.it (G.T.); michele.ghidini@policlinico.mi.it (M.G.)

**Keywords:** colorectal cancer, gastrointestinal malignancy, young-onset

## Abstract

The objective of this study is to fill the knowledge gap by examining predictors of lymph node metastasis (LNM) in young patients, less than 45 years, using a national cancer registry. Methods: Patients diagnosed with T1 colorectal cancer were identified in the Surveillance, Epidemiology, and End Results registry. In total, 692 patients with T1 colorectal cancer were identified. Most tumors occurred in white race (77.7%), between 40 and 44 years of age (49.4%), with grade III tumor differentiation (59.8%) and 1 to 1.9 cm size (32.2%), and were left-sided tumors (61.1%). The overall rate of LNM was 22.5% (*n* = 149). LNM was associated with tumor grade IV (undifferentiated) (odds ratio (OR) 2.94, CI: 1.06–8.12; *p* = 0.038), and increasing tumor size (1 cm–1.9 cm: OR 2.92, CI: 1.71–4.97, *p* < 0.001; 2.0 cm–2.9 cm: OR 2.00, CI: 1.05–3.77, *p* = 0.034; and ≥3.0 cm: OR 2.68, CI: 1.43–5.01, *p* = 0.002). Five-year cancer-specific survival for patients with LNM was 91% and for patients without LNM this was 98%. Adjusted cox proportion models showed that LNM was associated with a four times higher rate of mortality (hazard ratio (HR) 4.43, CI: 1.27–15.52, *p* = 0.020). In this population-based analysis of patients with T1 colorectal cancer, tumor size and grade were significant predictors of LNM.

## 1. Introduction

Colorectal cancer (CRC) is the third most diagnosed cancer around the world, with an incidence rate of 10.9% in men and 9.5% in women [1,2]. Increased surveillance of CRC has led to a decrease in incidence and mortality, and improvement in overall prognosis [3,4]. While prior reports have documented a decrease in the incidence of CRC in average risk adults, there is a growing incidence of CRC in younger patients under 44 years of age [5]. An estimated 37,148 newly diagnosed cases of CRC in young adults were documented between 1980 and 2016, with a growing incidence of 1.3% annually between 1996 and 2016 [6].

Stratification of CRC is guided in part by tumor-node-metastasis (TNM) as set forth by the American Joint Committee on Cancer (AJCC) [2,3]. A T1 CRC lesion is defined as a primary tumor invading only the submucosal layer, a T2 lesion has grown into the muscularis propria, a T3 lesion has grown through the muscularis propria and into the subserosa, and a T4 lesion has grown into the surface of the visceral peritoneum or has grown into or attached to other organs or structures [2,3].

Approximately 90% of T1 CRC lesions present as stage I and can be removed with endoscopic resection. T1 CRC with lymph node metastasis typically undergo surgical resection of the primary tumor and subsequent adjuvant chemotherapy. Additionally, T1 CRC lesions with stage IV rely on systemic chemotherapy, targeted drugs, or immune therapy [2,3,7,8,9]. Prior studies have studied predictors of lymph node metastasis in average risk adults [10]. However, studies on younger patients (less than 44 years) are lacking. The objective of this study is to fill the knowledge gap by examining and identifying predictors of lymph node metastasis (LNM) in young patients under 44 years of age with T1 CRC using a national cancer registry.

## 2. Materials and Methods

Patients less than 45 years of age diagnosed with T1 colorectal cancer between 2000 and 2016 were extracted from the Surveillance, Epidemiology and End Results (SEER) registry. Data were collected from 18 US registries, which, in aggregate, represent nearly 28% of the US population.

The demographic variables of interest were patient sex, age at diagnosis, race, and year of diagnosis. Age was categorized as 19–24 years, 25–29 years, 30–34 years, 35–39 years, and 40–44 years. Race was recorded as White, African American, American Indian/Alaska Native, and Asian or Pacific Islander. The year of diagnosis was treated as a continuous variable from 2000 to 2016. Pathological information included tumor size categorized as ≤9 mm, 1.0–1.9 cm, 2.0–2.9 cm, ≥3.0 cm, tumor stage, and tumor grade. The American Joint Committee on Cancer (AJCC) staging criteria was used. Tumor laterality was groups as left side (anorectum, sigmoid colon, descending colon), and right side (cecum, ascending colon, transverse colon). Tumor histology was classified as adenocarcinoma and mucinous type.

### Statistical Analysis

SEER*Stat statistical software (version 8.3.6; National Cancer Institute, Bethesda, MD, USA) was used to perform a case listing. Raw data were then exported and processed using IBM SPSS Statistics (version 25, Armonk, NY, USA). Logistic regression modelling was performed to determine predictors of LNM metastasis. Cox proportion hazard (PH) regression modeling was used to determine predictors of survival or mortality. Cox PH assumptions were evaluated by examining Schoenfeld residuals. Cox PH models were true if the hazard was reasonably constant over time. We excluded cases with unknown survival duration and statistical significance was set at *p*  <  0.05. SEER data are publicly available, de-identified, and exempt from institutional review board approval.

## 3. Results

### 3.1. Demographics and Tumor Characteristics

**Figure 1:**
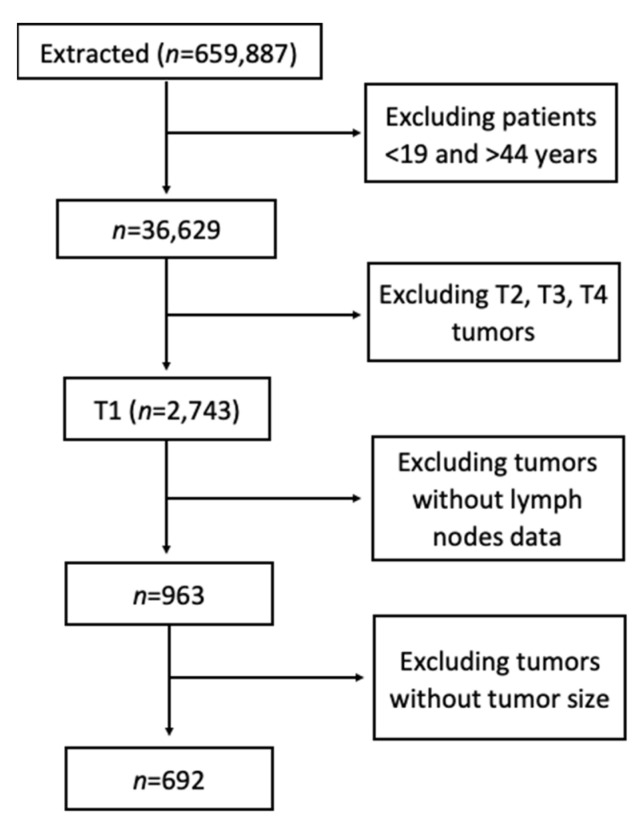
Study flow chart.

A total of 659,887 patients with colorectal cancer were identified of which 692 patients with stage T1 colorectal carcinoma were extracted (Figure 1). From this cohort, 53.9% were female and 77% were of White race. Most tumors on presentation were poorly differentiated (59.8%), followed by moderately differentiated (25.5%), well differentiated (8.2%), and undifferentiated (5.2%). Unknown tumor grade accounted for 1.3% of cases.

According to AJCC staging criteria, 77.5% was N0 and 22.5% was N1. Negative regional lymph nodes accounted for 78.5% of cases, while 21.5% of cases had positive lymph nodes. Most tumors occurred in the left side (61.1%) compared to the right side (33.4%). Analysis of tumor histology showed that most tumors were adenocarcinoma (75.4%), compared to mucinous type (2.6%). Unknown tumor histology accounted for 22% of cases (Table 1).

### 3.2. Predictors of Lymph Node Metastasis (LNM)

We evaluated predictors of LNM using univariate and multivariate models. Upon univariate analysis, sex, age, race, laterality, and tumor histology were not significant predictors of LNM. Univariate analysis showed that undifferentiated or grade IV tumors were about three times likely to have LNM (Odds ratio (OR) 2.66, Confidence Interval (CI): 1.04–6.81, *p* = 0.041); multivariate analysis showed a similar trend (OR 2.94, CI: 1.06–8.12, *p* = 0.038).

Univariate analysis also showed that increasing tumor size (>1cm) was a significant predictor of LNM. Compared to tumors less than 1 cm, tumor size of 1 cm to 1.9 cm were 3 times likely to have LNM (OR 2.92, CI: 1.74–4.89, *p* < 0.001), tumor size greater than 3 cm were 2.4 times likely to have LNM (OR 2.36, CI: 1.33–4.18, *p* = 0.003. On multivariate analysis, a similar trend was observed where increasing tumor size was associated with higher rates of LNM. Compared to tumors less than 1 cm, tumor size of 1 cm to 1.9 cm were 3 times likely to have LNM (OR 2.92, CI: 1.71–4.97, *p* < 0.001), tumor size 2 cm to 2.9 cm were 2 times likely to have LNM (OR 2.00, CI: 1.05–3.77, *p* = 0.034, and tumor size > 3 cm were about 3 times likely to have LNM (OR 2.68, CI: 1.43–5.01, *p* = 0.002). No other significant predictors were identified on multivariate analysis or stepwise logistic regression (Table 2).

### 3.3. Clinical Predictors of Mortality

On Cox PH regression analysis, higher mortality was associated with positive regional lymph nodes following univariate analysis (hazard ratio (HR) 5.45 CI: 1.73–17.18, *p* <0.004) and multivariate analysis (HR 4.43 CI: 1.27–17.52, *p* =0.020). Left-sided cancer was associated with 36% higher mortality; however, this was statistically insignificant (HR 0.64, CI: 0.38–1.08, *p* = 0.091) (Table 3).

**Figure 2:**
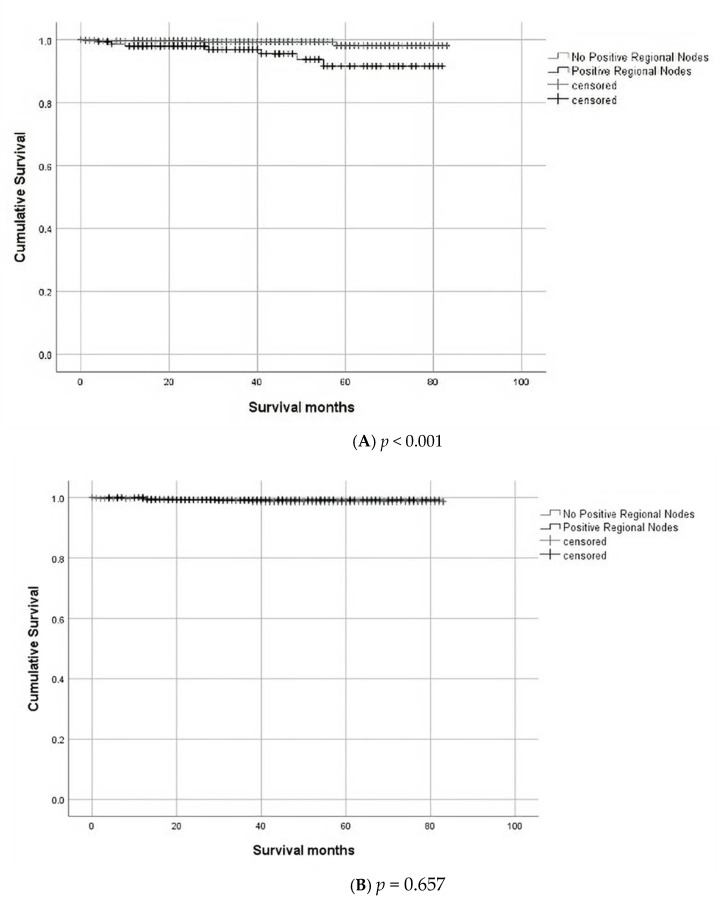
Kaplan Meier estimations showing the effect of lymph node metastasis on cancer specific mortality (**A**) and all cause mortality (**B**).

Kaplan Meier estimations were used to assess the impact of positive lymph node status on survival. Lymph node positivity did not affect all-cause mortality (log rank *p* = 0.657), however, positive lymph node status significantly affected cancer-specific mortality (log rank *p* < 0.001) (Figure 2). Three- and five-year survival rates for cancer-specific T1 CRC was 99% and 98% for patients without LNM, and 96% and 92% for patients with LNM.

## 4. Discussion

To date, this study represents the largest evaluation of young patients under 45 years with T1 colorectal cancer. In this population-based study, we investigated the predictors for LNM in T1 CRC in young patients. The accurate identification of predictors for LNM risk is crucial when distinguishing patients with low risk of LNM who can be treated using endoscopic resection rather than radical resection. Overall, the highest incidence of CRC was observed within the 40–44-year age-group and mildly higher in females. We found that tumor size and tumor grade were most predictive of lymph node metastasis.

In a retrospective study of 8056 patients over 18 years of age, it was found that the overall risk of LNM in patients with T1 colon cancer was 2% (*n* = 967), with tumor grade, tumor size, age, and race being identified as predictors of LNM [10]. Adjusted logistic regression models revealed that mucinous carcinoma (odds ratio (OR) = 2.26, *p* < 0.001), moderately differentiated (OR 1.74, *p* < 0.001), poorly differentiated (OR 5.16, *p* < 0.001), and undifferentiated carcinoma (OR 3.01, *p* = 0.003); older age (OR 0.66, *p* < 0.001 for age 65–79 years, OR 0.44, *p* < 0.001 for age over 80 years); and carcinoma located in the ascending colon (OR 0.77, *p* = 0.018) and sigmoid colon (OR 1.24, *p* = 0.014) were found to be independent predictive factors for LNM. Our study looked specifically at patients from 18 years to 44 years, and identified increasing tumor size and tumor grade as independent predictors of LNM. Unlike the prior study [10], age, race, gender, laterality, and tumor histology were not found to be independent predictors of LNM in young patients.

There are several well-known risk factors for CRC, such as obesity, high-fat diets, diets high in red meat, low physical activity, and low fiber intake. The risk factors for young-onset are unique. Inflammatory bowel disease is associated with a from two to three-fold increase in CRC and is more prevalent when seen in younger patients [11]. Known hereditary syndromes predisposed to CRC or family history of CRC is higher among young-onset [12]. Low adherence to cancer screening protocols for those with predispositions such as hereditary syndromes and family history of CRC [13]. Poor screening adherence is seen more in patients with low socioeconomic status. Race has also become a significant area of question, as there are clear disparities in survival and incidence reports between white and black patients [14,15]. Lastly, prior history of abdominal radiation exposure is typically seen in radiation therapy for pediatric malignancies [16].

Young-onset CRC have a higher occurrence of distinct epidemiological, histological, and initial presentation features as compared to older age groups [17]. In one study, there is a 32% occurrence of tumor in the rectum and progressive decreases in occurrence through older age groups. However, tumor occurrence in the cecum was 9.3% in the younger age group but progressively increased to 23.2% in the 85 and older age group [18]. Histologically, young-onset has a higher incidence of poorly differentiated, Mucinous and signet ring CRC compared to older groups, which typically present with adenocarcinoma CRC [19]. Younger onset of CRC is associated an increased risk of a secondary primary tumors within six months (synchronous tumor) (5.8% *vs.* 1.2%, *p =* 0.007) and risk of primary tumor after six months (metachronus tumor) (4.0% *vs.* 1.6%, *p =* 0.023) when compared to older groups [12] Lastly, and most clinically significant, a young onset has a lower 5 year survival, more advanced stage diseases are diagnosed, and higher incidence of reoccurrence and metastasis are found compared to older groups [17,20,21]

Inherited syndromes associated with CRC have been extensively studied. Syndromes commonly seen such as Lynch Syndrome (LS), Familial adenomatous polyposis (FAP), and Peutz–Jeghers syndrome (PJS), and many more have clear genes associated with each condition and clinical features to assist in diagnosis [22]. Lynch syndrome (LS), the most inherited CRC syndrome, currently encompasses 2–4% of all CRC cases [23]. Additionally, 70% of all individuals with LS develop CRC, which has led to significant strikes in the research of inherited risk factors for CRC [24]. The development of sporadic-mutation-associated CRC is less commonly studied.

There are currently no established mechanisms by which spontaneous young-onset CRC can arise, but microsatellite instability and CpG island methylation are the most studied. One study showed DNA from biopsy samples from sporadic young-onset CRC patients had a high frequency of mutation in PIK3R1, PDGFRA, FLT3, and KDR [25]. In another study, methylation of CpG islands as a mechanism in gene silencing was postulated as a potential driver of young onset CRC; CpG methylation has been observed in about 40% of all CRC cases [26,27].

Another unique feature of young-onset CRC is Line-1 hypomethylation [27]. In one observational study, a higher degree of LINE-1 hypomethylation was noted compared to older onset [28]. This was further supported by a separate cohort study, where they were able to show that young-onset CRC had significantly lower LINE-1 methylation than any other group (*p* < 0.0001) [26].

The current paradigm shows that, while there is overlap between adults >45 years with LNM and young adults <44 years, some factors remain unknown. As exemplified above, differences in tumor biology in this cohort may likely be responsible [5].

Our data show that patients with LNM have a higher risk of mortality, which has clinical and practical implications for patients. While these patients are often treated with endoscopy, these patients would benefit from a closer observation and follow up. Overwater et al. looked at 602 patients with T1 CRC with one or more histological risk factors for LNM who were treated with primary surgery or surgery following endoscopic resection [29]. Recurrence rates in the primary surgery group and in the secondary surgery group were 7.2% (19/263), and 4.8% (15/309), respectively. The overall recurrence rate for T1 CRC treated with primary surgery was 14.7 per 1000 person-years and was not statistically different from the overall recurrence rate for T1 CRC treated with secondary surgery, which was 9.7 per 1000 person–years (*p* = 0.297). In fact, more than half of recurrence cases involved distant metastasis after surgical treatment during long-term follow-up. These data indicate that despite mode of treatment (i.e., surgery vs endoscopy), patients with T1 disease remain at significant risk and should be closely monitored.

Cox regression models indicated that LNM was associated with five times higher mortality and poorer survival rates in patient with T1 CRC. These observations indicate the need to determine lymph node status in guiding therapeutic interventions. Patients with known risk factors for developing young onset CRC should receive more intensive cancer prevention visits.

In the present population-based analysis, our conclusions are based on a national cancer registry. Nevertheless, certain limitations must be acknowledged. The limited availability of data from the SEER database is the main drawback. Factors including submucosal invasion depth, tumor budding, and lymphovascular invasion might also affect the likelihood of LNM, which were not available for analysis. A large proportion of patients had unknown or missing tumor histology.

## 5. Conclusions

In conclusion, the overall LNM rate is approximately 22% for T1 CRC in young patients (less than 45 years). Tumor size and tumor grade are significant predictors for LNM in patients with T1 CRC cancer. Moreover, positive lymph node involvement is a significant prognostic factor for cancer specific survival. Thus, careful preoperative assessment of lymph node status is essential in clinical decision-making, to achieve better long-term outcomes.

## Figures and Tables

**Table 1:** Study characteristics.

Variables	Total	Percent
Sex		
Male	319	46.1%
Female	373	53.9%
Race		
White	538	77.7%
Black	85	12.3%
American Indian/Alaska Native	54	7.8%
Asian or Pacific Islander	7	1.0%
Unknown	8	1.2%
Age		
19–24 years	37	5.3%
25–29 years	57	8.2%
30–34 years	107	15.5%
35–39 years	149	21.5%
40–44 years	342	49.4%
N Staging (TNM)		
N0	536	77.5%
N1	156	22.5%
Regional Nodes		
Negative regional node	543	78.5%
Positive regional node	149	21.5%
Tumor Grade		
Well differentiated; Grade I	57	8.2%
Moderately differentiated; Grade II	176	25.5%
Poorly differentiated; Grade III	413	59.8%
Undifferentiated; anaplastic; Grade IV	36	5.2%
Unknown	10	1.4%
Tumor Size		
≤0.9 cm	197	28.5%
1 cm–1.9 cm	223	32.2%
2.0 cm–2.9 cm	130	18.8%
≥3.0 cm	142	20.5%
Laterality		
Left side	423	61.1%
Right side	231	33.4%
Unknown	38	5.5.%
Histology		
Adenocarcinoma	522	75.4%
Mucinous type	18	2.6%
Unknown	152	22.0%

**Table 2:** Logistic regression model showing predictors of lymph node metastasis.

	Multivariate			Univariate		
Characteristics	Odds Ratio	95% CI	*p*-Value	Odds Ratio	95% CI	*p*-Value
Sex						
Male	1.00			1.00		
Female	0.93	0.63–1.35	0.693	0.95	0.66–1.37	0.791
Age Group						
19–24 years	1.00			1.00		
25–29 years	2.10	0.67–6.64	0.548	2.08	0.68–6.38	0.198
30–34 years	1.52	0.50–4.58	0.206	1.38	0.48–4.01	0.552
35–39 years	2.21	0.75–6.47	0.461	2.06	0.75–5.67	0.163
40–44 years	2.02	0.71–5.72	0.149	1.80	0.68–4.77	0.239
Race						
White	1.00			1.00		
Black	1.20	0.68–2.10	0.527	1.16	0.67–1.99	0.604
Asian or Pacific Islander	1.29	0.66–2.52	0.461	1.31	0.69–2.50	0.406
American Indian/Alaska Native	1.86	0.33–10.36	0.480	1.50	0.29–7.84	0.630
Tumor Grade						
Well differentiated; Grade I	1.00			1.00		
Moderately differentiated; Grade II	1.04	0.47–2.31	0.922	0.97	0.45–2.06	0.927
Poorly differentiated; Grade III	1.24	0.58–2.66	0.579	1.15	0.57–2.31	0.697
Undifferentiated; anaplastic; Grade IV	2.94	1.06–8.12	0.038	2.66	1.04–6.81	0.041
Tumor Size						
≤0.9 cm	1.00			1.00		
1 cm–1.9 cm	2.92	1.71–4.97	<0.001	2.92	1.74–4.89	<0.001
2.0 cm–2.9 cm	2.00	1.05–3.77	0.034	1.80	0.983–3.30	0.057
≥3.0 cm	2.68	1.43–5.01	0.002	2.36	1.33–4.18	0.003
Laterality						
Left side	1.00			1.00		
Right side	0.64	0.38–1.08	0.091	0.81	0.54–1.20	0.288
Histology						
Adenocarcinoma	1.00			1.00		
Mucinous type	0.87	0.23–3.28	0.833	1.35	0.38–4.75	0.640
Unknown	2.37	1.18–4.74	0.015	1.51	0.41–5.51	0.534

**Table 3:** Cox regression model showing risk of mortality.

	Multivariate			Univariate		
Characteristics	Hazard Ratio	95% CI	*p*-Value	Hazard Ratio	95% CI	*p*-Value
Sex						
Male	1.00			1.00		
Female	0.58	0.18–1.89	0.365	0.61	0.19–1.9	0.396
Age Group						
19–24 years	1.00			1.00		
25–29 years	22,042	0.00–2.15 × 10^127^	0.945	9932.075	0.00–6.92 × 10^108^	0.940
30–34 years	5751.15	0.00–5.61 × 10^126^	0.952	4424.739	0.00–3.08 × 10^108^	0.946
35–39 years	7052.66	0.00–6.86 × 10^126^	0.951	10,214.540	0.00–7.08 × 10^108^	0.940
40–44 years	7385.87	0.00–7.18 × 10^126^	0.951	9932.075	0.00–6.76 × 10^108^	0.941
Race						
White	1.00			1.00		
Black	<0.01	0.00–3.45 × 10^12^	0.718	0.034	0.00–76.81	0.390
Asian or Pacific Islander	<0.01	0.00–1.03 × 10^19^	0.783	0.034	0.00–535.59	0.492
American Indian/Alaska Native	<0.01	0.00–0.00	0.982	0.034	0.00–3.2 × 10^10^	0.810
Regional Nodes						
Negative regional node	1.00			1.00		
Positive regional node	4.43	1.27–15.52	0.020	5.45	1.73–17.18	0.004
Tumor Grade						
Well differentiated; Grade I	1.00			1.00		
Moderately differentiated; Grade II	<0.01	0.00–2.04 × 10^54^	0.881	<0.01	0.00–5.01 × 10^216^	0.961
Poorly differentiated; Grade III	0.20	0.03–1.25	0.086	0.43	0.09–2.13	0.302
Undifferentiated; anaplastic; Grade IV	1.34	0.20–8.98	0.764	3.34	0.61–18.29	0.164
Tumor Size						
≤0.9 cm	1.00			1.00		
1 cm–1.9 cm	488.62	0.00–4.08 × 10^12^	0.595	55,877.89	0.00–3.00 × 10^96^	0.919
2.0 cm–2.9 cm	0.43	0.00–1.29 × 10^20^	0.972	1.01	0.00–2.83 × 10^139^	1.00
≥3.0 cm	633.04	0.00–5.31 × 10^12^	0.582	80,201.33	0.00–4.31 × 10^96^	0.917
Laterality						
Left side	1.00			1.00		
Right side	1.52	0.34–6.80	0.586	0.76	0.20–2.88	0.690
Histology						
Adenocarcinoma	1.00			1.00		
Mucinous type	4881.73	0.00–8.83 × 10^93^	0.981	25,990.41	0.00–1.44 × 10^234^	0.970

## Data Availability

Application for the SEER datasets can be made via https://seer.cancer.gov/ (accessed on 10 October 2021).
